# Microbial degradation of petrochemical waste-polycyclic aromatic hydrocarbons

**DOI:** 10.1186/s40643-017-0158-4

**Published:** 2017-06-30

**Authors:** M. H. Fulekar

**Affiliations:** 1 School of Environment and Sustainable Development, Central University of Gujarat, Sector 30, Gandhinagar, 382030 India; 2 Environmental Biotechnology Laboratory, Department of Life Sciences, University of Mumbai, Vidyanagari, Santacruz (E), Mumbai, 400098 India

**Keywords:** Microbial degradation, Polycyclic aromatic hydrocarbons, Microbial consortium, *Pseudomonas aeruginosa*

## Abstract

**Background:**

Petrochemical industry is one of the fastest growing industries. This industry has immense importance in the growth of economy and manufacture of large varieties of chemicals. The petrochemical industry is a hazardous group of industry generating hazardous waste containing organic and inorganic compounds. In spite of the present treatment process, the hazardous waste compounds are found untreated to the acceptable level and found discharged at soil–water environment resulting into the persistent organic–inorganic pollutant into the environment. The bioremediation will be the innovative techniques to remove the persistent pollutants in the environment.

**Result:**

Petrochemical contaminated site was found to be a rich source of microbial consortium degrading polycyclic aromatic hydrocarbons. Indigenous microbial consortiums were identified and used for bioremediation of polycyclic aromatic hydrocarbons (naphthalene and anthracene) at the concentrations of 250, 500, and 750 ppm. The potential microorganism was also identified for naphthalene and anthracene, and their bioremediation was studied at varying concentrations. The bioremediation with consortium was found to be comparatively more effective than the potential microorganism used for bioremediation of each compound.* Pseudomonas aeruginosa* a potential organism was identified by 16S rRNA and further studied for the gene responsible for the PAH compounds.

**Conclusion:**

Indigenous microorganism as a consortium has been found effective and efficient source for remediation of organic compound—Polycyclic aromatic hydrocarbon and this will also be applicable to remediate the toxic compounds to clean up the environment.

## Background

Petroleum is a complex mixture of hydrocarbons and other organic compounds, including some organometallo-constituents. Petroleum constituents represent: saturates, aromatics, resins, and asphaltenes. Saturates are defined as hydrocarbons containing no double bonds. They are categorized according to their chemical structures into alkanes (paraffins) and cycloalkanes. Saturates represents the highest percentage of crude oil constituents. The manufacture of petrochemicals begins with crude oil and natural gas which were formed millions of years ago, deep in the earth’s crust, due to the slow and lengthy decay of plants and animals. Worldwide industrial developments have released a large number of natural and synthetic hazardous compounds into the environment due to careless waste disposal, illegal waste dumping, and accidental spills. Industrial development has generated complex wastes, a complexity not only due to the quantity of wastes, but also to their composition (Wei and Huang 2001). As a result, there are numerous sites in the world that require clean-up of soils and sludge. Petroleum contamination of soil is a widespread and a well-recognized global environmental issue. One of the major constituents of petroleum is Polycyclic Aromatic Hydrocarbon’s (PAHs) which belong to the class of organic chemicals consisting of two or more benzene rings fused in a linear, angular, or cluster arrangement. PAHs are characterized by their high hydrophobicity, and resistance to natural degradation and carcinogenic properties. PAH releases to soils and other wider environment have led to higher concentrations of these contaminants that would not be expected from natural processes alone. They are known soil and aquatic contaminants. Whether naturally occurring or formed during the incomplete combustion of fossil fuels, low concentrations can usually be found just about everywhere. They are also associated with industrial activities and around wood-preservation stations where creosote oils have been used (Piskonen and Itävaara 2004).

Bioremediation is a promising option for the complete removal and destruction of contaminants. Bioremediation is the use of living organisms, primarily microorganisms, to degrade or detoxify hazardous wastes into harmless substances such as carbon dioxide, water, and cell biomass. As such, it uses relatively low-cost, low-technology techniques, which generally have a high public acceptance. PAHs are biodegradable (Da Silva et al. 2003; Meysami and Baheri 2003) and bioremediation for cleanup of PAH wastes has been extensively studied at both the laboratory and commercial levels, and has been implemented at a number of contaminated sites, including the well-published cleanup of the Exxon Valdez oil spill in Prince William Sound, Alaska in 1989; the Mega Borg spill-off the Texas coast in 1990; and the Burgan Oil Field, Kuwait in 1994 (Purwaningsih et al. 2002).

This research study has been designed to carry out bioremediation of PAH using indigenous microorganisms isolated from 3 decade old petrochemical waste disposal site. The petrochemical waste site was chosen for the study as the site is assumed to be rich in microorganisms adapted to extreme toxic organic waste and capable of adjusting to harsh environmental conditions. Microbial consortium and potential microorganism were isolated from the soil by enrichment technique in order to analyze the potential of the isolated strains to assimilate PAH under lab scale study under simulated conditions. The PAHs selected for this study were chosen on the basis of their extensive usage, toxicity, resistance to biodegradation, recalcitrant and persistent nature resulting in pollution. The compounds naphthalene and anthracene were selected for this bioremediation study. The soil collected was analyzed for physicochemical and microbial characteristics. The parameters indicated that the soil contained nutrients required for growth and proliferation of microorganisms. Microbial characterization was done by biochemical methods and 16S rRNA technique which showed the presence of various species of bacteria and fungi adapted to the harsh environment and survival in the contaminated soil.

The consortium utilized for bioremediation studies was isolated from the total culturable soil microbial population and identified by different morphological, physiological, biochemical assays, and 16S rRNA technology followed by BLAST. Bacterial consortium was enriched in Tanner’s Mineral Medium (TMM) and adapted to various concentrations of selected compounds viz. 250, 500, and 750 mg/L. Biodegradation studies were carried under controlled environmental conditions in shaker incubator for 14 days. The biodegrading capability of the consortium was assessed, and the potential pathway inducers, which are produced as intermediates during PAH degradation, have been identified by HPLC and GC/MS. The increase in the microbial count and the variation in pH, chemical oxygen demand (COD) and biological oxygen demand (BOD) were monitored and quantified as an indicator for growth and proliferation of microorganisms along the degradation of the selected PAHs in the mineral medium. The soil microbial consortium has been further assessed to isolate potential microorganism for biodegradation studies of the selected PAHs at concentrations of 250, 500, and 750 mg/L. The potential microorganism identified by 16S rRNA was found most similar to *Pseudomonas aeruginosa*. Further, the naphthalene catabolic gene has been targeted using PCR amplification, to analyze the presence of naphthalene-degrading gene in identified potential microorganism *P. aeruginosa* isolated from soil contaminated with aromatic hydrocarbons. The biodegrading ability of *P. aeruginosa* strain was confirmed by the presence of *NahAa* gene. The gene was present on a 25 kb plasmid isolated from *P. aeruginosa*. The amplified gene product of 337 bp was obtained using primers designer by PRIMER3 software and amplified by PCR technique. These microorganisms can be effectively used for bioremediation of PAHs contaminated wastes generated by petrochemical industries.

## Methods

### Site description and soil analysis

The petrochemical industry located at Khapri-Nagpur, Maharashtra (21.0495 °N, 79.0433 °E) have been disposing petrochemical waste effluent which contains a mixture of various PAH, motor oils, and grease dumped at this site through underground waste effluent channels since last 3 decades. Samples were collected aseptically from a layer 0–30-cm-deep site a few meters away from the petrochemical plant. The samples were collected in sterilized, sealed pack polythene bags which were later ground and sieved through a 2-mm pore size sieve and stored at 4 °C for further physicochemical and microbial assays for isolation of potential PAH-degrading microorganisms. The sample was used for microbial enumeration immediately after collection. For isolation of bacteria from the petrochemical contaminated soil/sediments, 1 g of the mixed soil was added to 9 mL of deionized water, and 0.1 mL of this diluted sample was spread by plating on Nutrient Agar medium from the appropriate dilution tubes and then incubated at the room temperature for 24 h. The plates showing isolated colonies were tallied, and the results were determined for each soil sample. The fungal colonies were counted after 48–72 h of incubation (Janbandhu and Fulekar 2009). Isolated colonies were plated on specific agars which were used for identifying specific microorganisms and fungi in the contaminated soil. Tanner’s Mineral Medium (MM) medium was used as a culture medium for enrichment and isolation of anthracene- and naphthalene-degrading strains. The composition of Tanner’s Mineral Medium (MM) medium was as follows (g/L): 0.04 CaCl_2_·H_2_O; 0.1 KH_2_PO_4_; 0.8 NaCl; 1.0 NH_4_Cl; 0.2 MgSO_4_·7H_2_O; 0.1 KCl. Micronutrients used were (mg/L) 0.1 CoCl_2_·6H_2_O; 0.425 MnCl_2_·4H_2_O; 0.05 ZnCl_2_; 0.015 CuSO_4_·5H_2_O; 0.01 NiCl_2_·6H_2_O; 0.01 Na_2_MoO_4_·2H_2_O; 0.01 Na_2_SeO_4_·2H_2_O. The pH of the medium was adjusted to 7.0.

### Enrichment and isolation of the anthracene- and naphthalene-degrading microbial consortium

The petrochemical industrial waste was expected to consist of microorganisms that adapted and survived at a higher contaminant concentration, and also has potential to degrade anthracene. The isolates were exposed to the increasing concentrations of anthracene and naphthalene separately to isolate the microorganisms that could utilize anthracene and naphthalene as sole carbon sources in the enrichment study by the method described by Siddique et al. (2003) using nutrient medium. The present bioremediation study was carried out by shake flask method. A quantity of fresh petrochemical contaminated soil was added into 100 mL sterilized MSM containing 250 mg/L of solid anthracene and naphthalene in a conical flask. Anthracene and naphthalene were added directly into MSM in solid state. Although this concentration was much higher than the real contamination level in environment, colonies were selected for further identification. All isolates were stored at −20 °C as liquid cultures containing 20% glycerol (v/v) for bioremediation studies.

After the first microbial enrichment in an orbital shaker (120 rpm) at 37 °C for 1 week, an aliquot of 5 mL enriched culture was inoculated into another 250-mL conical flask containing 100 mL fresh MSM with the same amounts of solid anthracene and naphthalene for the second enrichment. After four consecutive enrichments, the cultures with a series of concentration gradients were inoculated on the MSM agar plates containing thin layers of anthracene and naphthalene, respectively, to get the enriched consortium and the separated anthracene- and naphthalene-degrading microorganism with a clearing zone around the inoculated region. The isolation and purification of the bacterial consortium were carried out on nutrient agar plates by conventional spread plate techniques. Plates were incubated at 37 °C for 48 h after which isolated colonies were selected for further identification. All isolates were stored at −20 °C as liquid cultures containing 20% glycerol (v/v) (Zhao et al. 2009). Technique for isolation of microbial consortium capable of degrading PAH from petrochemical contaminated soil is given in flow-chart mentioned below.

#### Technique for isolation of microbial consortium capable of degrading PAH from petrochemical contaminated soil

**Table:** 

A quantity of fresh petrochemical contaminated soil was added into 100 mL sterilized TMM containing 250 mg/L solid naphthalene spiked in conical flask and shaken in an orbital shaker (150 rpm) at 37 °C for 1 week for microbial enrichment

After 7 days, an aliquot of 5 mL enriched culture was inoculated into another 250-mL conical flask containing 100 mL fresh TMM with the same amount of solid naphthalene used for the second enrichment

After four consecutive enrichments, 1 mL of culture was inoculated on the TMM agar plates containing a thin layer of naphthalene to get the enriched consortium and naphthalene-degrading microorganism were selected on the basis of clearing zone around the inoculated region

The cultures surviving at higher concentration were inoculated on the TMM agar plates containing a thin layer of Naphthalene on the agar to get the enriched consortium

The separated naphthalene-degrading microorganisms were identified by observing a clearing zone around the inoculated region

The isolation and purification of the individual colonies were carried out on nutrient agar plates by conventional spread plate technique

Plates were incubated at 37 °C for 48 h after which isolated colonies were selected for further identification. All isolates were stored at −20 °C as liquid cultures containing 20% glycerol (v/v) for bioremediation studies

### Identification of microbial consortium

The microbial isolates were first identified based on the morphological, cultural characteristics of individual colonies, then by traditional biochemical tests. Individual isolated colony was re-streaked on mineral agar plates for identification. The isolated colony was gram stained, and different standard morphological, physiological, and biochemical tests were performed using KB003 kit (KB003 Hi25, Himedia, India). Further the 16S rDNA gene sequence was used to carry out BLAST (Basic local Alignment Search tool) with ‘nr’ database of NCBI GenBank using MEGABLAST algorithm. The BLAST data were arranged in maximum percentage identity and first ten sequences was selected and exported in FASTA format. Based on maximum identity score and query coverage, the best highly identical 10 sequences were selected and aligned using multiple alignment software program, ClustalW (MEGA tool). The evolutionary history was inferred using the Neighbor-joining method. The bootstrap consensus tree inferred from 500 replicates is taken to represent evolutionary history of the taxa analyzed (Saitou and Nei 1987; Felsenstein 1985; Kimura 1980).

### Bioremediation experiment

#### Spiking of organic compound

Erlenmeyer flasks (250 mL) and Tanner’s Mineral Media were autoclaved for 20 min at 121 °C. 1 mL acetone containing the required anthracene and naphthalene concentrations was aseptically added to autoclaved Erlenmeyer flasks allowing the acetone to evaporate. After complete evaporation of acetone from the Erlenmeyer flasks, 100 mL sterile culture media was added under laminar flow hood so as to reach the desired final concentration of organic compound. The inocula containing 5% of the total volume were sampled in the logarithmic phase culture in MM broth and incubated for 24 h with orbital shaking (120 rpm). After incubation for 24 h, 1 mL medium was diluted, and 0.1 mL each of 107 dilutions was plated on nutrient agar and incubated for 24–30 °C in dark, and colonies were directly counted and expressed as CFU/mL. The pH was monitored throughout the bioremediation experiment.

#### Shake flask method

The biodegradation of selected PAH (anthracene and naphthalene) was performed individually in Borosil flask containing 100 mL of TMM inoculated with microbial consortium The experiment was carried out under controlled environmental conditions by continuously shaking at 150 rpm at 37 °C for 14 days The concentrations of organic compounds were monitored over time in order to compare lag periods, and biodegradation rates. At different concentrations, biodegradation was assessed by comparing the disappearance behaviors of organic compounds in samples compared with the controls over time, Samples (10 mL) were withdrawn hourly for up to 6 h, and afterward, at every 24 h over a period of 14 days for analysis by high-performance liquid chromatography (HPLC) and GC–MS. The environmental parameters like pH, temperature, colony forming units (CFUs), chemical oxygen demand (COD), and biological oxygen demand (BOD) were monitored throughout the experiment, Biodegradation was similarly carried out for all the three compounds with potential microorganism, *P. aeruginosa.*

**Table:** 

The biodegradation of selected PAHs (anthracene and naphthalene) was performed individually in Borosil flask containing 100 mL of Tanners Mineral Medium (TMM) inoculated with microbial consortium

The experiment was carried out under controlled environmental conditions by continuously shaking at 150 rpm at 37 °C for 14 days

The concentration of organic compounds were monitored over time in order to compare lag periods and biodegradation rates for different concentrations

Biodegradation was assessed by comparing the disappearance of organic compound in samples compared to the controls over time

Samples (10 mL) were withdrawn hourly for up to 6 h; and afterward at every 24 h over a period of 14 days for analysis by high-performance liquid chromatography (HPLC) and GC–MS

The environmental parameters like pH, temperature, colony forming units (CFU), chemical oxygen demand (COD) and biological oxygen demand (BOD) were monitored throughout the experiment

Biodegradation was similarly carried out for all the three compounds with potential microorganism *P. aeruginosa*

### Anthracene and naphthalene biodegradation

Samples were centrifuged (10 min, 10,000 rpm) to separate cell mass and the supernatant. The samples were extracted inorganic solvent (*n*-hexane) for analysis. Chromatographic separation was performed using HPLC system [Jasco, Model UV-2075Plus], equipped with the UV–VIS diode array detector (Varian) and with Borwin software. 20 µL of extracts was injected into the isocratic mobile phase of acetonitrile and water (80:20 ratios), run at 1 mL/min, isocratic run for 10 min with Varian C-18 column (250 mm × 4.6 mm). Anthracene and naphthalene were further identified by comparing UV spectra and retention times with the standards. Detections were performed at 254 nm. The extracts were also analyzed for identification of anthracene and naphthalene along with its metabolites by gas chromatography–mass spectrometry, and samples were quantified according to USEPA SW-846 Method 8270D (gas chromatography/mass spectrometry for semivolatile Organic compounds). The Shimadzu gas chromatograph (Model QT 2010) equipped with electron ionization detector and mass selective detector was used. The injector temperature was programmed from ambient to 360 °C where the oven temperature was fixed at 350 °C. Nitrogen was used as the carrier gas, and the temperature program was set as follows: 80 °C for 1 min, and then 5 °C/min up to 240 °C for 5 min. The degradation rate was estimated by calculating the decrease of the peak of the substrate PAH (Kimura 1980). Qualitative analysis was based on retention indexes, mass spectra comparison with data in the literature and mass spectral libraries, and comparison of the mass spectra to those of the commercially available compounds.

## Results and discussion

Petroleum industry started its operations in the year 1867 and is considered as the oldest Indian industry. India is one of the most flourishing oil markets in the world and, in the past few decades, has witnessed the expansion of top national companies producing a variety of chemicals beneficial to mankind in almost all sectors. Petrochemicals industry is a crucial member of the Indian economy since it caters to the needs of major industries like power, telecom, cables, plastics, textiles, etc. However, the wastes generated during manufacturing of raw materials and end products are of concern as they pose hazard to environment and living beings. The Hazardous Waste (management and handling) Rule (1989) has listed petrochemical industry as hazardous group of industries. Complex hazardous compounds are present in the effluent generated by petrochemical industries such as organic compounds, high and low molecular weight polycyclic aromatic hydrocarbons (PAH), heavy metals, oil, grease etc. which tend to persist in environment for longer period.

This study investigates the petrochemical contaminated site for isolation of microorganisms capable of assimilating PAH compounds mostly present at the petrochemical contaminate site. The microorganisms were isolated from the site as it was assumed that they would be able to survive in hazardous environment and assimilate selected hydrocarbons without inhibitory effect and would serve as a potential source for bioremediation process. The study highlights bioremediation of selected PAH compounds viz. naphthalene, and anthracene by microbial consortium as well as by potential microorganism by shake flask technique. Each of the selected compounds has been taken for bioremediation at varying concentrations (100, 250, and 500 mg/L) under controlled environmental conditions. The variations in environmental parameters such as COD, BOD, CFU, and pH have been monitored during bioremediation experiments. Intermediates formed during bioremediation have been detected for all the compounds to confirm degradation.

### Microbial assessment at petrochemical waste disposal site

The soil collected from the petrochemical contaminated site was analyzed for its microbial characteristics. The soil showed presence of macro and micronutrients which indicated that the soil supported growth of microorganisms even in PAH contaminated hazardous waste. Various heavy metals and organic compounds were detected in the soil. Analysis of Total Petroleum hydrocarbons (TPH) showed presence of 27 different organic compounds in the soil which mainly included saturated cycloalkanes, long-chain alkanes and PAHs.

Microbial analysis of soil showed presence of variety of bacteria and fungi viz. *Pseudomonas* sp., *Bacillus cereus*, *Bacillus licheniformis, Streptococci* sp., *Salmonella* sp., *E. coli* sp., *Alcaligen* sp., *Micrococcus* sp., *Aspergillus* sp., *Mucor* sp., *Penicillium* sp., *Rhizopus* sp., *Nocardia* sp., *Rhodococcus* sp. The microbial consortium employed for bioremediation of selected PAH compounds were isolated by enrichment technique and identified by 16S rRNA technique, and the sequences obtained were submitted to GenBank. The consortia included *Microbacterium* sp. (GenBank Accession No: JQ029161), *Sphingobacterium* sp. (GenBank Accession No: JQ029162), *Bacillus cereus* (GenBank Accession No: JF514501), *Bacillus licheniformis* (GenBank Accession No: JF514502) and a novel bacterium *Achromobacter insolitus* (GenBank Accession No: GQ334452.1). The potential microorganism *P. aeruginosa* was also isolated by selective enrichment technique and 16S rRNA amplification.

### Bioremediation of selected PAH’s by microbial consortium isolated from petrochemical contaminated soil

Biodegradation was carried for three compounds, viz. naphthalene, anthracene, and phenanthrene by shake flask method. Each of the selected compounds has been taken for bioremediation at varying concentrations (250, 500, and 750 mg/L) under controlled environmental conditions. Samples were withdrawn at regular intervals, 0–6 h on the 1st day followed by withdrawal of samples at every 24 h for 14 days. The amount biodegraded and the intermediates formed during biodegradation were analyzed by HPLC and GC–MS. The variations in environmental parameters (CFU, COD, BOD, and pH) which would be indicative of progress of biodegradation have been monitored during the experiment.

### Bioremediation of naphthalene using microbial consortium by shake flask method

Bioremediation of naphthalene was assessed using microbial consortium isolated from petrochemical contaminated soil at varying concentrations viz. 250, 500, 750 mg/L using TMM under controlled environmental conditions (150 rpm at 37 °C). Variations in parameters like CFU, COD, BOD, pH have been monitored throughout the bioremediation process along with detection of intermediates formed during bioremediation of naphthalene.

**Fig. 1:**
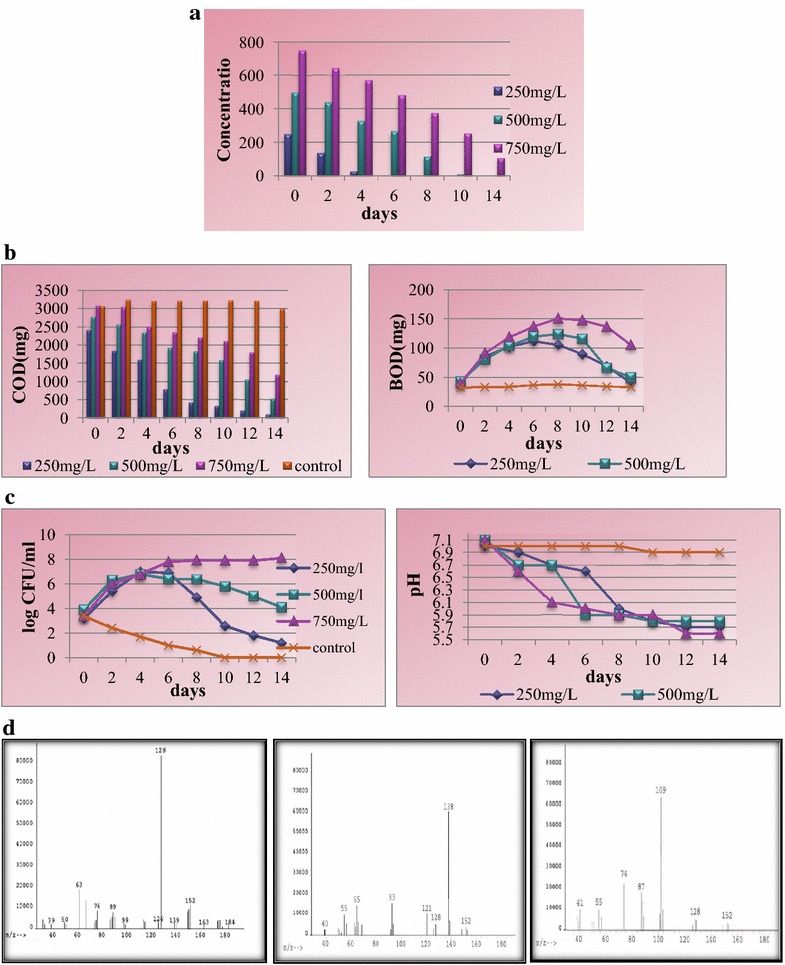
**a** Degradation of naphthalene by microbial consortium in TMM. **b** Variations in COD and BOD during bioremediation of naphthalene by microbial consortium in TMM. **c** Variations in viable count and pH during bioremediation of naphthalene by microbial consortium in TMM. **d** Mass spectra of naphthalene (*m/z* identification-128), salicylic acid (*m/z* identification-138), and catechol (*m/z* identification-109)

The interaction of microbial consortium with the contaminant led to degradation up to 750 mg/L without inhibitory effect on the consortium. Analysis of naphthalene bioremediation samples indicate that in the case of 250 mg/L naphthalene was completely degraded within 6 days followed by 500 mg/L of naphthalene which was degraded up to 98.4% within 10 days. At 750 mg/L naphthalene concentration, the compound was degraded up to 85.8% within 14 days (Fig. 1a).

In the case of 250 mg/L naphthalene, the CFU increased from the initial inoculated value followed by decline in the number of CFUs due to depletion of naphthalene from the minimal medium. At higher concentrations (500 and 750 mg/L) of naphthalene, the CFU gradually increased after which the growth ceased and entered the pseudo-stationary phase as the compound was not enough to maintain the growth of the population. Significant decrease in COD concentrations was observed at all the selected concentrations as the bioremediation experiment proceeded further (Fig. 1b). The initial values of BOD was almost the same for all the selected concentrations as the microorganisms inoculated into the minimal medium entered the lag phase due to the exposure to high concentration of compound (Fig. 1b). The BOD gradually increased during the biodegradation experiment for all the experimental initial concentrations, viz. 250, 500, and 750 mg/L of naphthalene. At lower concentrations, the BOD decreased due to depletion in the concentration of naphthalene. The change in pH was found to be shifting toward the acidic range for all the selected concentrations (Fig. 1c). The GC–MS analysis of the samples showed the presence of catechol and salicylic acid which were formed as intermediates during the bioremediation experiment (Fig. 1d).

### Bioremediation of anthracene using microbial consortium by shake flask method

**Fig. 2:**
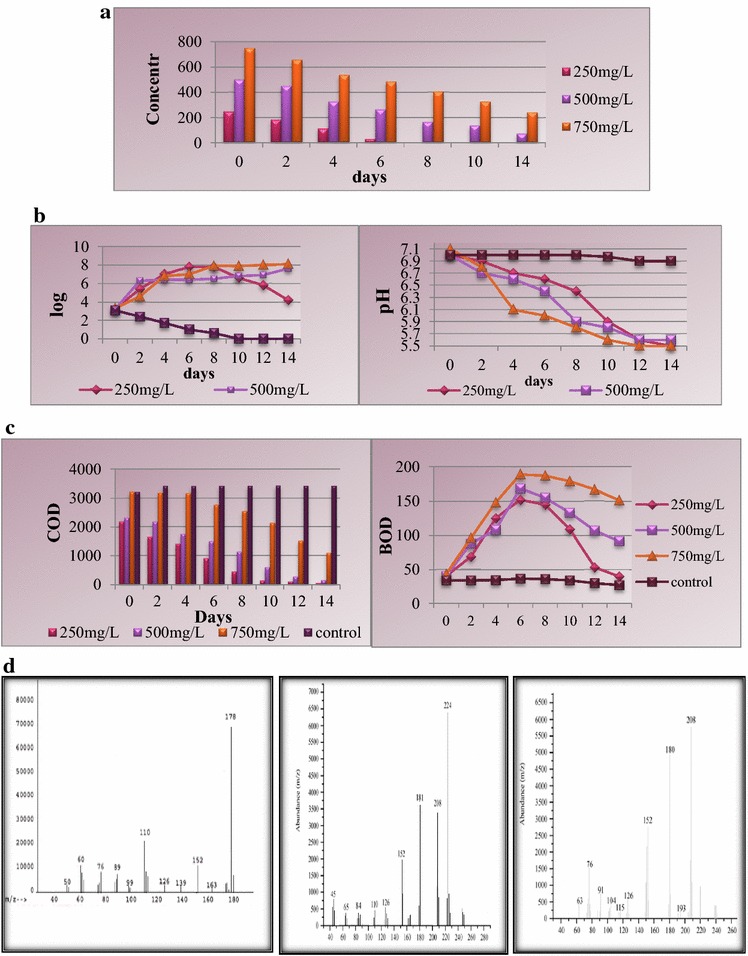
**a** Degradation of anthracene by microbial consortium in TMM. **b** Variation in viable count and variation in pH during bioremediation of anthracene by microbial consortium in TMM. **c** Variations in COD and BOD during bioremediation of anthracene by microbial consortium in TMM. **d** Mass spectra of Anthracene (*m/z* identification-178), 1-methoxy-2-hydroxy-anthracene (*m/z* identification-224), and Anthraquinone (*m/z* identification-208)

Biodegradation experiments were carried out using shake flask method for anthracene bioremediation at varying concentrations (250, 500, 750 mg/L) using microbial consortium isolated from petrochemical contaminated soil. The bioremediation of anthracene was carried out in TMM using anthracene as sole source of carbon. At the anthracene concentration of 250 mg/L, the compound was completely biodegraded within 8 days. At the concentration of 500 mg/L, the degradation was about 85.6% within a time span of 14 days. At the higher concentration of anthracene (750 mg/L), the biodegradation rate was higher than those for 250 and 500 mg/L of anthracene concentrations. About 67.8% of 750 mg/L anthracene was biodegraded within 14 days by the consortium (Fig. 2a). The CFU increased in the case of 250 mg/L of anthracene followed by a decrease in total cfu/mL due to depletion of the compound. In the cases of 500 and 750 mg/L of anthracene concentrations, the CFU increased from its initial value up to the fourteenth day as there was enough amount of anthracene in the mineral medium to allow for the growth of the microorganisms. The concentration of COD was found to be decreasing with the decreasing concentration of the organic contaminant for all the three concentrations, viz., 250, 500, and 750 mg/L of anthracene (Fig. 2b). BOD increased during the biodegradation experiment for all the experimental concentrations with the increasing CFU and decreased with decreasing CFU (Fig. 2c). At the anthracene concentrations of 250, 500, and 750 mg/L, BOD was found to be gradually increasing from the respective initial value after which it decreased as the concentration of anthracene depleted from the mineral medium. The change in pH was found to be shifting toward the acidic range due to the formation of acidic intermediates (Fig. 2c). The samples of each concentration were analyzed on a mass spectrometer for detection of intermediates formed during the bioremediation of anthracene. The mass spectrometer results showed the presence of 9,10-anthraquinone, 1-methoxy-2-hydroxy-anthracene, and 6,7-benzocoumarin as the major intermediates of anthracene biodegradation (Fig. 2d).

### Bioremediation of selected PAH’s compounds by potential microorganism *Pseudomonas aeruginosa*

Bioremediation of selected PAH’s was also studied by potential microorganism *P. aeruginosa*. The microorganism isolated from petrochemical contaminated soil using selective enrichment method was found to effectively degrade all the three compounds up to 750 mg/L. The bioremediation was carried out at varying concentrations viz. 250, 500, and 750 mg/L under controlled environmental conditions (150 rpm at 37 °C). Biodegradation was monitored for first 6 h for 1st day followed by every 24 h for 14 days. The progress of degradation and intermediates formed were analyzed by HPLC and GC–MS analysis. The variation in environmental parameters which included CFU, COD, BOD, and pH were monitored throughout the experiment.

### Bioremediation of naphthalene by potential microorganism *Pseudomonas aeruginosa*

**Fig. 3:**
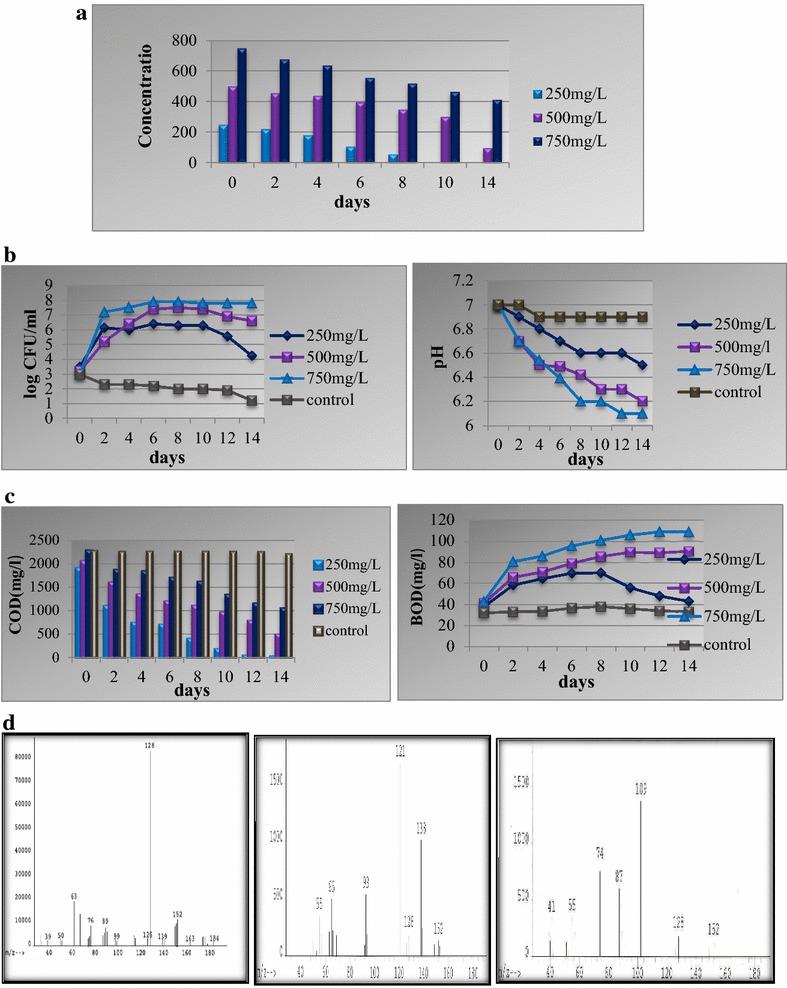
**a** Degradation of naphthalene by *Pseudomonas aeruginosa* in TMM. **b** Variations in viable count and pH during bioremediation of naphthalene by *P. aeruginosa* in TMM. **c** Variations in COD and BOD during bioremediation of naphthalene by *P. aeruginosa* in TMM. **d** Mass spectra of naphthalene (*m/z* identification 128), salicyliate (*m/z* identification 138), and catechol (*m/z* identification 109)

Naphthalene bioremediation was carried out by potential microorganism *P. aeruginosa* by shake flask method under controlled environmental conditions for 14 days. Analyses of naphthalene bioremediation samples indicate that in the case of 250 mg/L the compound was completely degraded within 10 days. At the 500 mg/L concentration of naphthalene, the compound was degraded to 80% within 14 days. At the higher naphthalene concentration (750 mg/L), about 45% of naphthalene was degraded by the potential microorganism *P. aeruginosa*. The essential environmental parameters that influence the rate of bioremediation were monitored throughout the experiment. In the case of 250 mg/L of naphthalene, the CFU increased for a few days, after which the growth ceased followed by a decrease in the total cfu/mL. In the cases of 500 and 750 mg/L naphthalene concentrations, the cfu/mL increased during the initial days after which the growth ceased and remained constant till the end of the experiment (Fig. 3a). COD was found to decrease with the decreasing concentration of the organic contaminant in TMM. Significant decreases in COD concentrations were observed at 250, 500, and 750 mg/L as the bioremediation experiment proceeded further (Fig. 3b). The Biological Oxygen Demand (BOD) was found to increase during the biodegradation experiment for all the experimental concentrations of naphthalene (Fig. 3b). At the naphthalene concentration of 250 mg/L, the initial concentration of BOD gradually increased for a week, after which there was decrease in the BOD concentrations, till the end of the experiment. At 500 and 750 mg/L naphthalene concentrations, BOD was found to be gradually increasing from its initial value up to the fourteenth day. The pH was found to be shifting toward the acidic range from initial neutral value due to the formation of acidic intermediates (Fig. 3c). The bioremediation samples were analyzed on mass spectrometer for detection of the intermediates formed during bioremediation. The mass spectrometry analysis of the samples showed the presence of catechol and salicylate during the bioremediation experiment (Fig. 3d).

### Bioremediation of anthracene by potential microorganism *Pseudomonas aeruginosa*

**Fig. 4:**
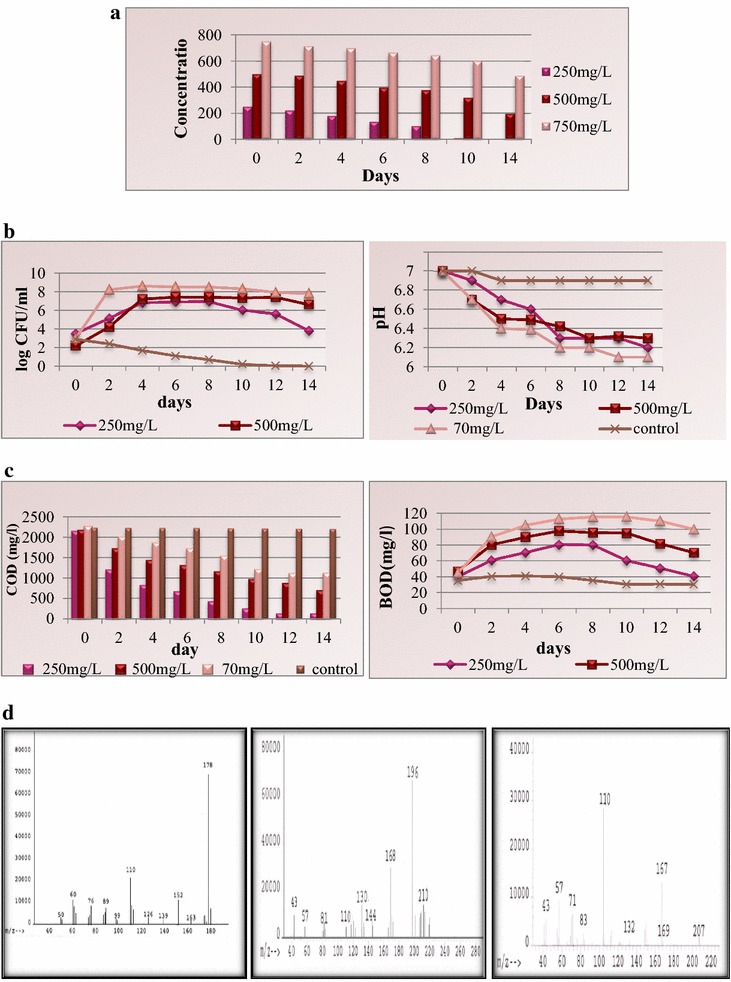
**a.**Degradation of anthracene by *Pseudomonas aeruginosa* in TMM. **b** Variations in viable count and pH during bioremediation of anthracene by *P. aeruginosa* in TMM. **c** Variations in COD and BOD during bioremediation of anthracene by *P. aeruginosa* in TMM. **d** Mass spectrum of Anthracene (*m/z* identification-178), benzocoumarin (*m/z* identification-196, 168, 139), and catechol (*m/z* identification-110)

Bioremediation of anthracene was carried out by shake flask at concentrations of 250, 500, and 750 mg/L using potential microorganism *P. aeruginosa*. At the anthracene concentration of 250 mg/L, the compound was completely biodegraded within 14 days. At the concentration of 500 mg/L, the degradation was about 60.8% within time span of 14 days. At the higher concentration of anthracene (750 mg/L), about 35% was biodegraded within 14 days by the consortium. The essential environmental parameters that influence the rate of bioremediation were monitored throughout the experiment. In the case of 250 mg/L of anthracene, the CFU increased initially after which growth ceased for few days followed by decrease in total cfu/mL as the experiment proceeded. In the cases of 500 and 750 mg/L anthracene concentrations, the cfu/mL increased initially for few days and remained constant till end of the experiment (Fig. 4a). The decrease in COD was observed at all the selected concentration with increase in time duration (Fig. 4b). The Biological Oxygen Demand (BOD) values were found to increase during the biodegradation experiment for all the experimental concentrations of anthracene except for 250 mg/L where the BOD decreased after a week as the compound was completely utilized (Fig. 4b). The pH was found to be shifting toward the acidic range from initial neutral value due to formation of acidic intermediates during bioremediation (Fig. 4c). The samples of each concentration were analyzed on mass spectrometer for detection of intermediated formed during the bioremediation of anthracene. The mass spectrometer results showed the presence of 6,7-Benzocoumarin, 2,3-dihydroxynaphthalene, and catechol as major intermediates (Fig. 4d).

### Comparison of bioremediation of naphthalene and anthracene by microbial consortium and potential microorganism *Pseudomonas aeruginosa*

Bioremediation of selected PAH has been carried out using microbial consortium and potential microorganism independently by shake flask method under controlled environmental conditions. The biodegradation of these hazardous aromatic compounds differ as they have different chemical structure. The bioremediation studies carried out for selected organic compounds, viz. naphthalene and anthracene using microbial consortium as well as potential microorganism *P. aeruginosa*, have been compared. Bioremediation study of naphthalene shows that the microbial consortium and potential microorganism were effective in biodegrading naphthalene at all the three concentrations viz. 250, 500, and 750 mg/L, but the rates and concentrations of degradation of the three selected compounds were higher in the case of microbial consortium.

The bioremediation results showed that the consortium was able to biodegrade the selected organic compounds efficiently up to 750 mg/L. At the 250 mg/L of naphthalene concentration, the microbial consortium degraded 100% of the compound within 6 days, and the potential microorganism degraded 100% of the compound within 10 days. Similarly, at 500 mg/L concentration, the microbial consortium degraded 98.4% within 10 days, and the potential microorganism degraded 80.7% within 14 days. At the higher concentration of 750 mg/L, about 85.8% of naphthalene was degraded and 45% by potential microorganism within 14 days.

In a similar manner, bioremediation studies for anthracene show that for 250 mg/L, the degradation reached 100% within 8 days by microbial consortium and 100% within 14 days by potential microorganism. At 500 mg/L, about 85.6% was degraded by microbial consortium and 60.8% was degraded by potential microorganism. At 750 mg/L, the microbial consortium assimilated 67.8%, whereas potential microorganism assimilated 35% of the compound within 14 days.

The biodegradation studies for naphthalene illustrated that at 250 mg/L concentration, 98% was degraded within 8 days and 100% by potential microorganism within 14 days. At 500 mg/L, about 81.5% was degraded by microbial consortium, and 58.9% was degraded by potential microorganism. At 750 mg/L, the microbial consortium assimilated 63.5%, whereas potential microorganism assimilated 34.7% of the compound within 14 days (Table 1).

**Table 1:** Comparison of % biodegradation of naphthalene and anthracene by microbial consortium and potential microorganism

Organic contaminant (%)	0 day		4th day		8th day		10th day		14th day	
	Microbial consortium	*Pseudomonas aeruginosa*	Microbial consortium	*Pseudomonas aeruginosa*	Microbial consortium	*Pseudomonas aeruginosa*	Microbial consortium	*Pseudomonas aeruginosa*	Microbial consortium	*Pseudomonas aeruginosa*
250 mg/L (% degradation)										
Naphthalene	1.5	0.5	89.5	28.5	–	78	–	99	–	–
Anthracene	0.7	0	42.4	20.8	100	60	–	96	–	100
500 mg/L (% degradation)										
Naphthalene	2.4	0	34.5	12.5	76.7	30.7	98.4	40	–	80.7
Anthracene	1.4	0	29.6	10.5	66.7	24.8	74.2	36.5	85.6	60.8
750 mg/L (% degradation)										
Naphthalene	3.3	0	23.5	15	49.8	30.9	66.3	38	85.8	45
Anthracene	2.1	0	23.5	7	45.8	14.2	56.4	20	67.8	35

The comparison demonstrated that naphthalene was degraded higher than anthracene followed by phenanthrene in both the cases, i.e., by consortium and potential microorganism. Though the compound anthracene was degraded at faster rate than phenanthrene, but the difference in amount degraded was not major. This is because both anthracene and phenanthrene have same chemical structure with three benzene rings which are positioned in a linear and skewed manner. The order of biodegradation of these selected PAH compounds by consortium and potential microorganism was found to be naphthalene > anthracene. Although the potential microorganism *P. aeruginosa* was capable of biodegrading all the selected organic compounds up to 750 mg/L, the microbial consortium degraded the selected compounds more efficiently and rapidly than the potential microorganism at all the selected concentrations (Table 1).

Naphthalene being a linear 2 ring compound was degraded faster followed by anthracene which is a linear three-ring compound. Since naphthalene and anthracene are linear in structure, the initial attack of the microbes on PAH is in the K and bay regions of the compound. Furthermore, studies have reported that synergistic effects or enhancement of degradation efficiency in consortia can result from (1) cooperative effects due to different complementary biochemical PAH-degradation pathways in the strains (Wang et al. 2006); (2) assimilation of PAH onto cell membrane and changes of cell hydrophobicity by excreted substances such as biosurfactants (Phale and Mahajan 1995; Prabhu and Phale 2003; Andreoni et al. 2004); (3) elimination of PAH metabolites by one strain that inhibits other strains (Bouchez Naïtali et al. 1999); and (4) significant increment of biomass using surfactants as a primary carbon source (Kim and Weber 2003).

### Identification of gene responsible for naphthalene degradation in potential microorganism *Pseudomonas aeruginosa*

Despite its low solubility in water naphthalene is frequently encountered in effluents in complex mixtures like petroleum fractions, creosote, and pharmaceutical wastes (Mueller 1989). Biological method of treatment has turned out to be a favorable alternative for naphthalene degradation, and several reports are available on the removal of naphthalene by different microorganisms (Grund et al. 1992). Biological methods include bioremediation of the hazardous organic compound by potential microorganisms which are capable of utilizing the contaminant as carbon and energy source. The catabolic property of microorganisms to utilize the contaminant as carbon and energy source is conveyed by the genes present in the potential microorganism, which encode for specific enzymes in catabolic pathways. In the present research study, *P. aeruginosa* was found efficient in assimilating naphthalene as the sole source of carbon and energy. Hence, the further aim of this research study was to identify the gene responsible for naphthalene biodegradation by PCR-amplification technique.

In the aerobic biodegradation of PAHs, dioxygenases are the key enzymes. Naphthalene dioxygenase (NDO) is the first enzyme for the degradation of naphthalene in various bacterial strains (Eaton and Chapman 1992) and is able to catalyze the dioxygenation of more than 60 different aromatic compounds (Resnick et al. 1996). Each enzyme consists of three components, a ferredoxin reductase (*NahAa*), a ferredoxin (*NahAb*), and an iron–sulfur protein composed of large and small subunits, α (*NahAc*) and β (*NahAd*), respectively (Kauppi 1998), and the substrate specificity of *NDO* is primarily determined by the α-subunit (Parales et al. 1999). In this study, the occurrence of the naphthalene dioxygenase (*NahAa*) gene which catalyzes the first step in the degradation of naphthalene genes in the isolate was analyzed to confirm its catabolic potential.

**Fig. 5:**
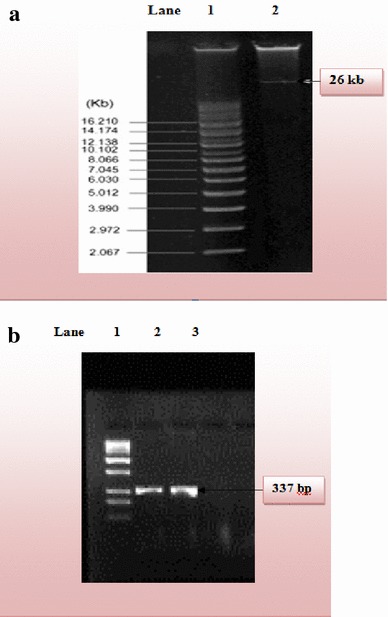
**a** Genomic characterization of potential microorganism responsible for bioremediation Anthracene. Plasmid DNA isolated from potential microorganism, *Pseudomonas aeruginosa. Lane 1* DNA ladder, *Lane 2* 26 kb plasmid. **b** Agarose gel of PCR products. *Lane 1* (from *left*) 1-kb ladder, *lane 2* 337-bp DNA fragment generated from *P. putida* NCIB 9816-4 which was taken as positive control, and *lane 3* 337-bp DNA fragment generated from potential microorganism *P. aeruginosa* strain using *nahAa* probe

Catabolic pathways, which encode different aromatic hydrocarbon degradation routes, are frequently located on either chromosome or plasmid. In the specific case of PAHs, genes responsible for degradation are likely to be located on plasmids. Therefore, the possible involvement of catabolic plasmids in naphthalene degradation by *P. aeruginosa* strain was investigated. Electrophoretic separation profile of plasmid DNA isolated from *P. aeruginosa* culture showed that it contained one plasmid of 26 kb size (Fig.5a). (Coral and Karagoz 2005) also reported isolation of 26 kb plasmid from *Pseudomonas* sp. The plasmid DNA isolated from *P. aeruginosa* was used for *NahAa* gene amplification. Through catabolic gene identification, it was found that the plasmid DNA of Gram-negative naphthalene-degrading bacteria *P. aeruginosa* strain contained conserved ferredoxin reductase (*NahAa*) gene. Amplification was done by primers designed by PRIMER3 software. The primer pair successfully amplified *NahAa* gene fragment of expected size from plasmid DNA. The amplified product of 337 bp was obtained, and the sequence was determined (Fig. 5b). The nucleotide sequence obtained has been submitted to the NCBI GenBank under accession number JQ029163. BLAST result showed that the gene was 100% similar to reductase component *NahAa* of naphthalene dioxygenase gene of *Pseudomonas putida* (GenBank accession no. YP_534820.1), 99% similar to ferredoxin reductase gene of *Burkholderia* sp., C3 (GenBank Accession No. ACT53245.1) and 98% similar to reductase component of salicylate 5-hydroxylase of *Achromobacter xylosoxidans* (GenBank Accession No. YP_195873.1). Presence of ferredoxin reductase gene (*NahAa*) and GC–MS results confirmed naphthalene biodegradation by salicylic acid pathway. This gene has also been characterized quantitatively by Cho and Tiedje (2002) and Tsuda and Lino (1990). Thus, the PCR technique with the *NahAa* marker gene can conveniently be used in the goal-directed search for naphthalene-degrading microorganisms in soil. Characterization of the genes, which encode degradative activities, may contribute to the evaluation of microbial populations optimal for biodegradation and bioremediation technologies.

The research study signifies the potential of microorganisms isolated from petrochemical contaminated soil. Both consortium and potential microorganism have been proved to be effective in biodegradation of petrochemical hydrocarbons. This bioremediation technology established by developing potential and effective sources of microbial consortium and potential microorganism would be applicable for treatment of effluent/waste to decontaminate the hazardous PAH compounds from the contaminated soil and waste water and would be a cost-effective method in the cleaning-up process of the environment.

## Conclusions

The present research study investigates potential of microorganisms isolated from petrochemical-contaminated soil for bioremediation of PAH compounds. PAH-contaminated soil is the rich source of PAH-degrading microbes as the microorganisms isolated from the soil effectively degraded the selected PAH compounds. This research study has shown effective biodegradations of naphthalene and anthracene up to 750 mg/L by microbial consortium and potential microorganism *P. aeruginosa* by shake flask method under controlled environmental conditions. The highest degradation was accomplished for naphthalene followed by anthracene by the consortium consisting of five microorganisms. viz. *Microbacterium* sp., *Sphingobacterium* sp., *Bacillus cereus*, *Bacillus licheniformis*, and a novel bacterium *Achromobacter insolitus*.

The consortium used for biodegradation was found to be more efficient in the biodegradation of selected PAH compounds compared with potential microorganism *P. aeruginosa*. This ability to biodegrade the PAH more effectively can be attributed to the cooperative synergistic effect due to different complementary biochemical PAH-degradation pathways in the strains present in the consortium. The consortium and potential microorganism displayed degradation of the selected aromatic compounds that are likely to be found in the sites contaminated with complex mixtures of PAHs, indicating the possibility of using the consortium by increasing the population of these microorganisms at the sites contaminated with mixtures of polynuclear aromatic hydrocarbons for efficient biodegradation. The potential PAH-degrading microorganism *P. aeruginosa* has been isolated and identified by 16S rRNA technique (GenBank Accession No: JN609593) and was found to more effectively degrade the naphthalene than was anthracene up to 750 mg/L. Hence, the gene responsible for naphthalene degradation present in *P. aeruginosa* was further identified. The gene was found on a 26-kb plasmid isolated from potential microorganism and was partially amplified using target-specific primers. The amplified sequence has been submitted to NCBI (GenBank Accession No: JQ029163). A more complete understanding of the degradation genes as well as of their respective roles in their natural environment could provide a basis for the development of future bioremediation strategies. The potential microorganism can be used for efficient removal of hazardous waste contaminated with PAH compounds generated by petrochemical industries.

## References

[CR1] Andreoni V (2004). Bacterial communities and enzyme activities of PAHs polluted soils. Chemosphere.

[CR2] Bouchez Naïtali M, Rakatozafy H, Marchal R, Leveau JY, Vandecasteele JP (1999). Diversity of bacterial strains degrading hexadecane in relation to the mode of substrate uptake. J Appl Microbiol.

[CR3] Cho JC, Tiedje JM (2002). Quantitative detection of microbial genes by using DNA microarrays. Appl Environ Microbiol.

[CR4] Coral G, Karagoz S (2005). Isolation and characterization of phenanthrene-degrading bacteria from a petroleum refinery soil. Ann Microbiol.

[CR5] Da Silva M, Cerniglia CE, Pothuluri JV, Canhos VP, Esposito E (2003). Screening filamentous fungi isolated from estuarine sediments for the ability to oxidize polycyclic aromatic hydrocarbons. World J Microbiol Biotechnol.

[CR6] Eaton RW, Chapman PJ (1992). Bacterial metabolism of naphthalene: construction and use of recombinant bacteria to study ring cleavage of 1,2-dihydroxynaphthalene and subsequent reactions. J Bacteriol.

[CR7] Felsenstein J (1985). Confidence limits on phylogenies: an approach using the bootstrap. Evolution.

[CR8] Grund E, Denecke B, Eichenlaub R (1992). Naphthalene degradation via salicylate and gentisate by *Rhodococcus* sp. strain B4. Appl Environ Microbiol.

[CR9] Janbandhu A, Fulekar MH (2009). Characterization of PAH contaminated soil for isolation of potential microorganism capable of degrading naphthalene. Biosci Biotechnol Res Asia.

[CR10] Kauppi Björn (1998). Structure of an aromatic-ring-hydroxylating dioxygenase–naphthalene 1, 2-dioxygenase. Structure.

[CR11] Kim Han S, Weber Walter J (2003). Preferential surfactant utilization by a PAH-degrading strain: effects on micellar solubilization phenomena. Environ Sci Technol.

[CR12] Kimura M (1980). A simple method for estimating evolutionary rate of base substitutions through comparative studies of nucleotide sequences. J Mol Evol.

[CR13] Meysami P, Baheri H (2003). Pre-screening of fungi and bulking agents for contaminated soil bioremediation. Adv Environ Res.

[CR14] Mueller JG, Chapman PH, Pritchard PH (1989). Creosote-contaminated sites. Their potential for bioremediation. Environ Sci Technol.

[CR15] Parales JV, Parales RE, Gibson DT (1999). Aspartate 205 in the catalytic domain of naphthalene dioxygenase is essential for activity. J Bacteriol.

[CR16] Phale PS, Mahajan MC (1995). Vaidyanathan CS A pathway for degradation of 1- naphthoic acid by *Pseudomonas maltophilia* CSV89. Arch Microbiol.

[CR17] Piskonen R, Itävaara M (2004). Evaluation of chemical pretreatment of contaminated soil for improved PAH bioremediation. Appl Microbiol Biotechnol.

[CR18] Prabhu Y, Phale PS (2003). Biodegradation of phenanthrene by *Pseudomonas* sp. strain PP2: novel metabolic pathway, role of biosurfactant and cell surface hydrophobicity in hydrocarbon assimilation. Appl Microbiol Biotechnol.

[CR19] Purwaningsih IS, Hill GA, Headley JV (2002). Air stripping and dissolution rates of aromatic hydrocarbon particles in a bioreactor. Chem Eng Commun.

[CR20] Resnick SM, Lee K, Gibson DT (1996). Diverse reactions catalyzed by naphthalene dioxygenase from *Pseudomonas* sp. strain NCIB 9816. J Ind Microbiol.

[CR21] Saitou N, Nei M (1987). The neighbor-joining method: a new method for reconstructing Phylogenetic trees. J Mol Biol Evol.

[CR22] Siddique T, Okeke BC, Arshad M, Frankenberger WT (2003). Enrichment and isolation of endosulfan-degrading microorganisms. J Environ Qual.

[CR23] Tsuda M, Iino T (1990). Naphthalene degrading genes on plasmid NAH7 are on a defective transposon. Mol Gen Genet.

[CR24] Wang Z, Zhang J, Zhang Y, Hesham AL, Yang M (2006). Molecular characterization of a bacterial consortium enriched from an oilfield that degrades phenanthrene. Biotech Lett.

[CR25] Wei MS, Huang KH (2001). Recycling and reuse of industrial wastes in Taiwan. Waste Manage.

[CR26] Zhao HP, Wu QS, Wang L, Zhao XT, Gao HW (2009). Degradation of phenanthrene by bacterial strain isolated from soil in oil refinery fields in Shanghai China. J Hazard Mater.

